# Supplementary material for: Association Between Genomic Features and Radiation Response in Metastatic Breast Cancer Patients Undergoing Palliative Radiotherapy
Source: Int J Mol Sci. 2025 Dec 8;26(24):11837. doi: 10.3390/ijms262411837 (PMC12732987; doi:10.3390/ijms262411837)
Supplement: Supplementary file 1 [file ijms-26-11837-s001.zip › ijms-3984462-supplementary.pdf]

Table S1. List of 320 mutated genes and 791 mutation events identified in metastatic breast cancer.

Gene	Number of Mutations
TP53	23
PIK3CA	15
FAT1	14
ZFHX3	11
BRCA2	9
PRKDC	9
ANKRD11	7
ARID1B	7
ERBB3	7
FANCA	7
GATA3	7
KEL	7
LRP1B	7
MAP3K4	7
MDC1	7
POLE	7
RANBP2	7
SPTA1	7
ESR1	6
HOXB13	6
MGA	6
RECQL4	6
ROS1	6
SPEN	6
ASXL2	5
ATRX	5
CARD11	5
CDH1	5
FANCD2	5
FLT1	5
ICOSLG	5
MET	5
NAB2	5
NF1	5
NOTCH1	5
NUTM1	5
PIK3C2B	5
PREX2	5
ALK	4

ATM	4
CSF1R	4
EP300	4
EPCAM	4
ERBB2	4
FGFR2	4
GNAS	4
HGF	4
HSD3B1	4
IGF1R	4
INSR	4
MAGI2	4
MPL	4
MST1	4
NCOR1	4
NOTCH4	4
NTRK3	4
PDGFRA	4
PDGFRB	4
PTEN	4
PTPRS	4
SETD2	4
ALOX12B	3
ANKRD26	3
AR	3
ARID1A	3
ASXL1	3
BARD1	3
BCOR	3
BCORL1	3
BLM	3
BRCA1	3
DNMT1	3
EMSY	3
EPHB1	3
FANCG	3
FANCI	3
FLI1	3
FOXP1	3
GEN1	3
HIST1H1C	3

HSP90AA1	3
JAK1	3
JAK3	3
KMT2A	3
MAP3K1	3
MYCN	3
NOTCH3	3
NRG1	3
NSD1	3
PARP1	3
PAX7	3
PIK3R1	3
RAD52	3
RET	3
RPS6KA4	3
SETBP1	3
SH2B3	3
SLX4	3
TBX3	3
TCF3	3
TSC1	3
ZNF703	3
ABL1	2
ABL2	2
AMER1	2
ATR	2
AXIN1	2
BCL6	2
BCR	2
BIRC3	2
BRAF	2
BRD4	2
BRIP1	2
CASP8	2
CD74	2
CHD2	2
CHD4	2
CSF3R	2
CTCF	2
CTNNA1	2
DAXX	2

DDX41	2
DIS3	2
ERBB4	2
ERCC1	2
ERCC5	2
ETS1	2
ETV1	2
ETV6	2
FANCL	2
FGFR1	2
FGFR3	2
FGFR4	2
FLT3	2
FLT4	2
FOXL2	2
FUBP1	2
GID4	2
IGF2	2
IL7R	2
IRS1	2
KAT6A	2
KDM5A	2
KDM6A	2
MALT1	2
MAP2K2	2
MAP3K13	2
MCL1	2
MLH1	2
MLLT3	2
MRE11	2
MSH6	2
MTOR	2
NBN	2
NKX2-1	2
NOTCH2	2
NTRK1	2
PAK3	2
PALB2	2
PBRM1	2
PGR	2
PIK3R2	2

PLCG2	2
PMS1	2
PPM1D	2
PRSS8	2
PTPRD	2
PTPRT	2
RARA	2
RNF43	2
RPTOR	2
RUNX1	2
SLIT2	2
SMAD2	2
SMC3	2
SMO	2
SNCAIP	2
STAG1	2
STAG2	2
SYK	2
TAF1	2
TET1	2
TFE3	2
TMEM127	2
TRAF7	2
TSHR	2
VEGFA	2
ZBTB2	2
ZNF217	2
ADGRA2	1
AKT1	1
AKT3	1
APC	1
ARAF	1
ARID2	1
AXIN2	1
AXL	1
B2M	1
BCL2L2	1
BTG1	1
BTK	1
CALR	1
CBFB	1

CBL	1
CCND2	1
CD276	1
CDC73	1
CDK12	1
CDK4	1
CDKN2A	1
CHEK2	1
CIC	1
CREBBP	1
CSNK1A1	1
CTLA4	1
CTNNB1	1
CUX1	1
DDR2	1
DNMT3B	1
DOT1L	1
E2F3	1
EPHA3	1
EPHA5	1
EPHA7	1
ERCC2	1
ERCC3	1
ETV4	1
EWSR1	1
FAM46C	1
FANCC	1
FANCF	1
FAS	1
FH	1
FLCN	1
FOXA1	1
FYN	1
GABRA6	1
GATA1	1
GATA6	1
GLI1	1
GRM3	1
H3F3C	1
HIST1H2BD	1
HIST1H3E	1

HIST1H3G	1
HIST1H3H	1
HIST3H3	1
ID3	1
IDH2	1
IKBKE	1
IL10	1
IRF4	1
IRS2	1
JUN	1
KEAP1	1
KIF5B	1
KLF4	1
KRAS	1
LATS2	1
LYN	1
LZTR1	1
MAP2K4	1
MAP3K14	1
MAPK3	1
MED12	1
MSH2	1
MST1R	1
MTOR	1
MUTYH	1
MYB	1
MYOD1	1
NCOA3	1
NEGR1	1
NFE2L2	1
NPM1	1
NUP93	1
PAK1	1
PDK1	1
PHF6	1
PIK3C2G	1
PIK3CD	1
PLK2	1
PNRC1	1
POLD1	1
PRDM1	1

PRKN	1
PTCH1	1
RAD21	1
RAD50	1
RAD54L	1
RASA1	1
RB1	1
REL	1
RPS6KB1	1
RPS6KB2	1
SDHA	1
SDHC	1
SDHD	1
SMAD3	1
SOCS1	1
SOX17	1
SOX9	1
STAT4	1
STK11	1
SUFU	1
TCF7L2	1
TFRC	1
TGFBR1	1
TGFBR2	1
TNFRSF14	1
TOP1	1
TOP2A	1
TRAF2	1
TSC2	1
U2AF1	1
VTCN1	1
XIAP	1
XPO1	1
YES1	1
ZRSR2	1

Table S2. List of 141 altered pathways with corresponding pathway size, number of samples with pathway alteration, and contributing genes in metastatic breast cancer patient cohort.

Pathway	Number of Samples with Pathway Alteration	Pathway Size	Overlap Genes
Central carbon metabolism in cancer	24	71	AKT1, AKT3, ERBB2, FGFR1, FGFR2, FGFR3, FLT3, IDH2, KRAS, MAP2K2, MAPK3, MET, MTOR, NTRK1, NTRK3, PDGFRA, PDGFRB, PDK1, PIK3CA, PIK3CD, PIK3R1, PIK3R2, PTEN, RET, TP53
PI3K-Akt signaling pathway	23	362	AKT1, AKT3, BRCA1, CCND2, CDK4, CSF1R, CSF3R, ERBB2, ERBB3, ERBB4, FGFR1, FGFR2, FGFR3, FGFR4, FLT1, FLT3, FLT4, HGF, HSP90AA1, IGF1R, IGF2, IL7R, INSR, IRS1, JAK1, JAK3, KRAS, MAGI2, MAP2K2, MAPK3, MCL1, MET, MTOR, MYB, NTRK1, PDGFRA, PDGFRB, PIK3CA, PIK3CD, PIK3R1, PIK3R2, PTEN, RET, RPS6KB1, RPS6KB2, RPTOR, STK11, SYK, TP53, TSC1, TSC2, VEGFA
Breast cancer	23	148	JUN, AKT1, AKT3, APC, ARAF, AXIN1, AXIN2, BRAF, BRCA1, BRCA2, CDK4, CSNK1A1, CTNNB1, E2F3, ERBB2, ESR1, FGFR1, FLT4, IGF1R, KRAS, MAP2K2, MAPK3, MTOR, NCOA3, NOTCH1, NOTCH2, NOTCH3, NOTCH4, PGR, PIK3CA, PIK3CD, PIK3R1, PIK3R2, PTEN, RB1, RPS6KB1, RPS6KB2, TCF7L2, TP53
Prostate cancer	22	106	AKT1, AKT3, AR, ARAF, BRAF, CREBBP, CTNNB1, E2F3, EP300, ERBB2, FGFR1, FGFR2, HSP90AA1, IGF1R, KRAS, MAP2K2, MAPK3, MTOR, PDGFRA, PDGFRB, PIK3CA, PIK3CD, PIK3R1, PIK3R2, PTEN, RB1, TCF7L2, TP53
Non-small cell lung cancer	22	73	AKT1, AKT3, ALK, ARAF, BRAF, CDK4, CDKN2A, E2F3, ERBB2, HGF, JAK3, KIF5B, KRAS, MAP2K2, MAPK3, MET, PIK3CA, PIK3CD, PIK3R1, PIK3R2, PLCG2, RB1, RET, TP53
EGFR tyrosine kinase inhibitor resistance	21	80	AKT1, AKT3, ARAF, AXL, BRAF, ERBB2, ERBB3, FGFR2, FGFR3, HGF, IGF1R, JAK1, KRAS, MAP2K2, MAPK3, MET, MTOR, NF1, NRG1, PDGFRA, PDGFRB, PIK3CA, PIK3CD,

			PIK3R1, PIK3R2, PLCG2, PTEN, RPS6KB1, RPS6KB2, VEGFA
Endocrine resistance	21	99	JUN, AKT1, AKT3, ARAF, BRAF, CDK4, CDKN2A, E2F3, ERBB2, ESR1, GNAS, IGF1R, KRAS, MAP2K2, MAPK3, MTOR, NCOA3, NCOR1, NOTCH1, NOTCH2, NOTCH3, NOTCH4, PIK3CA, PIK3CD, PIK3R1, PIK3R2, RB1, RPS6KB1, RPS6KB2, TP53
Melanoma	20	73	AKT1, AKT3, ARAF, BRAF, CDH1, CDK4, CDKN2A, E2F3, FGFR1, HGF, IGF1R, KRAS, MAP2K2, MAPK3, MET, PDGFRA, PDGFRB, PIK3CA, PIK3CD, PIK3R1, PIK3R2, PTEN, RB1, TP53
Colorectal cancer	19	87	JUN, AKT1, AKT3, APC, ARAF, AXIN1, AXIN2, BRAF, CTNNB1, KRAS, MAP2K2, MAPK3, MLH1, MSH2, MSH6, MTOR, PIK3CA, PIK3CD, PIK3R1, PIK3R2, RPS6KB1, RPS6KB2, SMAD2, SMAD3, TCF7L2, TGFBR1, TGFBR2, TP53
Pancreatic cancer	19	77	AKT1, AKT3, ARAF, BRAF, BRCA2, CDK4, CDKN2A, E2F3, ERBB2, JAK1, KRAS, MAPK3, MTOR, PIK3CA, PIK3CD, PIK3R1, PIK3R2, RB1, RPS6KB1, RPS6KB2, SMAD2, SMAD3, TGFBR1, TGFBR2, TP53, VEGFA
Platinum drug resistance	18	75	AKT1, AKT3, ATM, BIRC3, BRCA1, CASP8, CDKN2A, ERBB2, ERCC1, FAS, MAPK3, MLH1, MSH2, MSH6, PIK3CA, PIK3CD, PIK3R1, PIK3R2, TOP2A, TP53, XIAP
MAPK signaling pathway	18	300	JUN, AKT1, AKT3, ARAF, BRAF, CSF1R, DAXX, ERBB2, ERBB3, ERBB4, FAS, FGFR1, FGFR2, FGFR3, FGFR4, FLT1, FLT3, FLT4, HGF, IGF1R, IGF2, INSR, KRAS, MAP2K2, MAP2K4, MAP3K1, MAP3K13, MAP3K14, MAP3K4, MAPK3, MET, NF1, NTRK1, PAK1, PDGFRA, PDGFRB, RASA1, RET, RPS6KA4, TGFBR1, TGFBR2, TP53, TRAF2, VEGFA
Glioma	18	76	AKT1, AKT3, ARAF, BRAF, CDK4, CDKN2A, E2F3, IGF1R, KRAS, MAP2K2, MAPK3, MTOR, PDGFRA, PDGFRB, PIK3CA, PIK3CD, PIK3R1, PIK3R2, PLCG2, PTEN, RB1, TP53

Gastric cancer	18	150	AKT1, AKT3, APC, ARAF, AXIN1, AXIN2, BRAF, CDH1, CSNK1A1, CTNNA1, CTNNB1, E2F3, ERBB2, FGFR2, HGF, KRAS, MAP2K2, MAPK3, MET, MLH1, MTOR, PIK3CA, PIK3CD, PIK3R1, PIK3R2, RB1, RPS6KB1, RPS6KB2, SMAD2, SMAD3, TCF7L2, TGFBR1, TGFBR2, TP53
Proteoglycans in cancer	17	204	AKT1, AKT3, ARAF, BRAF, CBL, CTNNB1, ERBB2, ERBB3, ERBB4, ESR1, FAS, FGFR1, HGF, IGF1R, IGF2, KRAS, MAP2K2, MAPK3, MET, MTOR, PAK1, PIK3CA, PIK3CD, PIK3R1, PIK3R2, PLCG2, PTCH1, RPS6KB1, RPS6KB2, SMAD2, SMO, TP53, VEGFA
Endometrial cancer	17	59	AKT1, AKT3, APC, ARAF, AXIN1, AXIN2, BRAF, CDH1, CTNNA1, CTNNB1, ERBB2, KRAS, MAP2K2, MAPK3, MLH1, PIK3CA, PIK3CD, PIK3R1, PIK3R2, PTEN, TCF7L2, TP53
Ras signaling pathway	16	238	ABL1, ABL2, AKT1, AKT3, CSF1R, ETS1, FGFR1, FGFR2, FGFR3, FGFR4, FLT1, FLT3, FLT4, HGF, IGF1R, IGF2, INSR, KRAS, MAP2K2, MAPK3, MET, NF1, NTRK1, PAK1, PAK3, PDGFRA, PDGFRB, PIK3CA, PIK3CD, PIK3R1, PIK3R2, PLCG2, RASA1, REL, VEGFA
Thyroid hormone signaling pathway	16	122	AKT1, AKT3, CREBBP, CTNNB1, EP300, ESR1, KRAS, MAP2K2, MAPK3, MED12, MTOR, NCOA3, NCOR1, NOTCH1, NOTCH2, NOTCH3, NOTCH4, PIK3CA, PIK3CD, PIK3R1, PIK3R2, PLCG2, TP53, TSC2
MicroRNAs in cancer	16	320	ABL1, APC, ATM, BCL2L2, BRCA1, CCND2, CDKN2A, CREBBP, DNMT1, DNMT3B, E2F3, EP300, ERBB2, ERBB3, FGFR3, FOXP1, IRS1, IRS2, KRAS, MAP2K2, MAPK3, MCL1, MET, MTOR, NOTCH1, NOTCH2, NOTCH3, NOTCH4, PDGFRA, PDGFRB, PIK3CA, PIK3CD, PIK3R1, PIK3R2, PLCG2, PTEN, RPTOR, SOCS1, TP53, VEGFA
Hepatocellular carcinoma	16	170	AKT1, AKT3, APC, ARAF, ARID1A, ARID1B, ARID2, AXIN1, AXIN2, BRAF, CDK4, CDKN2A, CSNK1A1, CTNNB1, E2F3, HGF, IGF1R, IGF2, KEAP1, KRAS, MAP2K2, MAPK3, MET, MTOR, NFE2L2, PBRM1, PIK3CA, PIK3CD, PIK3R1, PIK3R2, PLCG2, PTEN, RB1, RPS6KB1, RPS6KB2, SMAD2, SMAD3, TCF7L2, TGFBR1, TGFBR2, TP53

Longevity regulating pathway	15	90	AKT1, AKT3, IGF1R, INSR, IRS1, IRS2, KRAS, MTOR, PIK3CA, PIK3CD, PIK3R1, PIK3R2, RPS6KB1, RPS6KB2, RPTOR, STK11, TP53, TSC1, TSC2
Human papillomavirus infection	15	333	AKT1, AKT3, APC, ATM, ATR, AXIN1, AXIN2, CASP8, CCND2, CDK4, CHD4, CREBBP, CSNK1A1, CTNNB1, EP300, FAS, GNAS, IKBKE, JAK1, KRAS, MAP2K2, MAPK3, MTOR, NOTCH1, NOTCH2, NOTCH3, NOTCH4, PDGFRB, PIK3CA, PIK3CD, PIK3R1, PIK3R2, PTEN, RB1, RPS6KB1, RPS6KB2, TCF7L2, TP53, TSC1, TSC2, VEGFA
Chronic myeloid leukemia	15	77	ABL1, AKT1, AKT3, ARAF, BCR, BRAF, CBL, CDK4, CDKN2A, E2F3, KRAS, MAP2K2, MAPK3, PIK3CA, PIK3CD, PIK3R1, PIK3R2, RB1, RUNX1, SMAD3, TGFBR1, TGFBR2, TP53
Small cell lung cancer	15	93	AKT1, AKT3, BIRC3, CDK4, E2F3, PIK3CA, PIK3CD, PIK3R1, PIK3R2, PTEN, RB1, TP53, TRAF2, XIAP
Cellular senescence	14	157	AKT1, AKT3, ATM, ATR, CCND2, CDK4, CDKN2A, CHEK2, E2F3, ETS1, KRAS, MAP2K2, MAPK3, MRE11, MTOR, NBN, PIK3CA, PIK3CD, PIK3R1, PIK3R2, PTEN, RAD50, RB1, SMAD2, SMAD3, TGFBR1, TGFBR2, TP53, TSC1, TSC2
Renal cell carcinoma	14	70	JUN, AKT1, AKT3, ARAF, BRAF, CREBBP, EP300, ETS1, FH, FLCN, HGF, KRAS, MAP2K2, MAPK3, MET, PAK1, PAK3, PIK3CA, PIK3CD, PIK3R1, PIK3R2, TFE3, VEGFA
Apoptosis	13	137	JUN, AKT1, AKT3, ATM, BIRC3, CASP8, DAXX, FAS, KRAS, MAP2K2, MAP3K14, MAPK3, MCL1, NTRK1, PARP1, PIK3CA, PIK3CD, PIK3R1, PIK3R2, TP53, TRAF2, XIAP
Signaling pathways regulating pluripotency of stem cells	13	144	AKT1, AKT3, APC, AXIN1, AXIN2, CTNNB1, FGFR1, FGFR2, FGFR3, FGFR4, ID3, IGF1R, JAK1, JAK3, KAT6A, KLF4, KRAS, MAP2K2, MAPK3, PIK3CA, PIK3CD, PIK3R1, PIK3R2, SMAD2, SMAD3, TBX3, TCF3, ZFHX3
Human T-cell leukemia virus 1 infection	13	224	JUN, AKT1, AKT3, ATM, ATR, B2M, CALR, CCND2, CDK4, CDKN2A, CHEK2, CREBBP, E2F3, EP300, ETS1, JAK1, JAK3, KRAS, MAP2K2, MAP2K4, MAP3K1, MAP3K14, MAPK3, PIK3CA, PIK3CD, PIK3R1, PIK3R2, PTEN, RB1, SMAD2, SMAD3, TCF3, TGFBR1, TGFBR2, TP53, XIAP, XPO1

ErbB signaling pathway	13	86	JUN, ABL1, ABL2, AKT1, AKT3, ARAF, BRAF, CBL, ERBB2, ERBB3, ERBB4, KRAS, MAP2K2, MAP2K4, MAPK3, MTOR, NRG1, PAK1, PAK3, PIK3CA, PIK3CD, PIK3R1, PIK3R2, PLCG2, RPS6KB1, RPS6KB2
FoxO signaling pathway	12	133	AKT1, AKT3, ARAF, ATM, BCL6, BRAF, CCND2, CREBBP, EP300, IGF1R, IL10, IL7R, INSR, IRS1, IRS2, KRAS, MAP2K2, MAPK3, PIK3CA, PIK3CD, PIK3R1, PIK3R2, PLK2, PTEN, SMAD3, STK11, TGFBR1, TGFBR2
Sphingolipid signaling pathway	12	122	AKT1, AKT3, FYN, KRAS, MAP2K2, MAPK3, PIK3CA, PIK3CD, PIK3R1, PIK3R2, PTEN, TP53, TRAF2
Neurotrophin signaling pathway	12	120	JUN, ABL1, AKT1, AKT3, BRAF, IRS1, KRAS, MAP2K2, MAP3K1, MAPK3, NTRK1, NTRK3, PIK3CA, PIK3CD, PIK3R1, PIK3R2, PLCG2, SH2B3, TP53
Fanconi anemia pathway	12	55	ATR, BLM, BRCA1, BRCA2, BRIP1, ERCC1, FANCA, FANCC, FANCD2, FANCF, FANCG, FANCI, FANCL, MLH1, PALB2, SLX4
Rap1 signaling pathway	11	212	AKT1, AKT3, BRAF, CDH1, CSF1R, CTNNB1, FGFR1, FGFR2, FGFR3, FGFR4, FLT1, FLT4, GNAS, HGF, IGF1R, INSR, KRAS, MAGI2, MAP2K2, MAPK3, MET, PDGFRA, PDGFRB, PIK3CA, PIK3CD, PIK3R1, PIK3R2, VEGFA
Hepatitis B	11	163	JUN, AKT1, AKT3, ARAF, BRAF, CASP8, CREBBP, E2F3, EP300, FAS, IKBKE, JAK1, JAK3, KRAS, MAP2K2, MAP2K4, MAP3K1, MAPK3, PIK3CA, PIK3CD, PIK3R1, PIK3R2, RB1, SMAD3, STAT4, TGFBR1, TGFBR2, TP53
Longevity regulating pathway - multiple species	11	62	AKT1, AKT3, IGF1R, INSR, IRS1, IRS2, KRAS, MTOR, PIK3CA, PIK3CD, PIK3R1, PIK3R2, RPS6KB1, RPS6KB2, RPTOR
Acute myeloid leukemia	11	68	AKT1, AKT3, ARAF, BRAF, CSF1R, FLT3, KRAS, MAP2K2, MAPK3, MTOR, PIK3CA, PIK3CD, PIK3R1, PIK3R2, RARA, RPS6KB1, RPS6KB2, RUNX1, TCF7L2
Homologous recombination	10	41	ATM, BARD1, BLM, BRCA1, BRCA2, BRIP1, MRE11, NBN, PALB2, POLD1, RAD50, RAD52, RAD54L
p53 signaling pathway	10	75	ATM, ATR, CASP8, CCND2, CDK4, CDKN2A, CHEK2, FAS, PPM1D, PTEN, TP53, TSC2

PD-L1 expression and PD-1 checkpoint pathway in cancer	10	90	JUN, AKT1, AKT3, ALK, JAK1, KRAS, MAP2K2, MAPK3, MTOR, PIK3CA, PIK3CD, PIK3R1, PIK3R2, PTEN, RPS6KB1, RPS6KB2
Focal adhesion	10	203	JUN, AKT1, AKT3, BIRC3, BRAF, CCND2, CTNNB1, ERBB2, FLT1, FLT4, FYN, HGF, IGF1R, MAPK3, MET, PAK1, PAK3, PDGFRA, PDGFRB, PIK3CA, PIK3CD, PIK3R1, PIK3R2, PTEN, VEGFA, XIAP
Human cytomegalovirus infection	10	227	AKT1, AKT3, B2M, CALR, CASP8, CDK4, CDKN2A, CTNNB1, E2F3, FAS, GNAS, JAK1, KRAS, MAP2K2, MAPK3, MTOR, PDGFRA, PIK3CA, PIK3CD, PIK3R1, PIK3R2, RB1, RPS6KB1, RPS6KB2, TP53, TRAF2, TSC1, TSC2, VEGFA
Choline metabolism in cancer	10	99	JUN, AKT1, AKT3, KRAS, MAP2K2, MAPK3, MTOR, PDGFRA, PDGFRB, PIK3CA, PIK3CD, PIK3R1, PIK3R2, RPS6KB1, RPS6KB2, TSC1, TSC2
Kaposi sarcoma-associated herpesvirus infection	10	196	JUN, AKT1, AKT3, CASP8, CDK4, CREBBP, CTNNB1, E2F3, EP300, FAS, IKBKE, JAK1, KRAS, LYN, MAP2K2, MAP2K4, MAPK3, MTOR, PIK3CA, PIK3CD, PIK3R1, PIK3R2, PLCG2, RB1, SYK, TCF7L2, TP53, TRAF2, VEGFA
HIF-1 signaling pathway	10	110	AKT1, AKT3, CREBBP, EP300, ERBB2, FLT1, IGF1R, INSR, MAP2K2, MAPK3, MTOR, PDK1, PIK3CA, PIK3CD, PIK3R1, PIK3R2, PLCG2, RPS6KB1, RPS6KB2, TFRC, VEGFA
Growth hormone synthesis, secretion and action	10	122	AKT1, AKT3, CREBBP, EP300, GNAS, IRS1, IRS2, KRAS, MAP2K2, MAP2K4, MAP3K1, MAPK3, MTOR, PIK3CA, PIK3CD, PIK3R1, PIK3R2, PLCG2, SOCS1
Measles	9	139	JUN, AKT1, AKT3, CASP8, CCND2, CDK4, FAS, IKBKE, JAK1, JAK3, PIK3CA, PIK3CD, PIK3R1, PIK3R2, TP53
Hepatitis C	9	159	AKT1, AKT3, ARAF, BRAF, CASP8, CDK4, CTNNB1, E2F3, FAS, IKBKE, JAK1, KRAS, MAP2K2, MAPK3, PIK3CA, PIK3CD, PIK3R1, PIK3R2, RB1, TP53, TRAF2
Thyroid cancer	9	37	BRAF, CDH1, CTNNB1, KRAS, MAP2K2, MAPK3, NTRK1, RET, TCF7L2, TP53
Bladder cancer	9	41	ARAF, BRAF, CDH1, CDK4, CDKN2A, E2F3, ERBB2, FGFR3, KRAS, MAP2K2, MAPK3, RB1, TP53, VEGFA

Transcriptional misregulation in cancer	9	201	ATM, BCL6, BIRC3, CCND2, CSF1R, DOT1L, ETV1, ETV4, ETV6, EWSR1, FLI1, FLT1, FLT3, IGF1R, KDM6A, KMT2A, MET, MLLT3, MYCN, NCOR1, NTRK1, PAX7, RARA, REL, RUNX1, TCF3, TFE3, TGFBR2, TP53
B cell receptor signaling pathway	9	91	JUN, AKT1, AKT3, BTK, CARD11, KRAS, LYN, MALT1, MAP2K2, MAPK3, PIK3CA, PIK3CD, PIK3R1, PIK3R2, PLCG2, SYK
Fc epsilon RI signaling pathway	9	69	AKT1, AKT3, BTK, FYN, KRAS, LYN, MAP2K2, MAP2K4, MAPK3, PIK3CA, PIK3CD, PIK3R1, PIK3R2, PLCG2, SYK
Fluid shear stress and atherosclerosis	8	142	JUN, AKT1, AKT3, CTNNB1, HSP90AA1, KEAP1, MAP2K4, NFE2L2, PIK3CA, PIK3CD, PIK3R1, PIK3R2, TP53, VEGFA
VEGF signaling pathway	8	60	AKT1, AKT3, KRAS, MAP2K2, MAPK3, PIK3CA, PIK3CD, PIK3R1, PIK3R2, PLCG2, VEGFA
Type II diabetes mellitus	8	47	INSR, IRS1, IRS2, MAPK3, MTOR, PIK3CA, PIK3CD, PIK3R1, PIK3R2, SOCS1
Regulation of lipolysis in adipocytes	8	59	AKT1, AKT3, GNAS, INSR, IRS1, IRS2, PIK3CA, PIK3CD, PIK3R1, PIK3R2, TSHR
Progesterone-mediated oocyte maturation	7	111	AKT1, AKT3, ARAF, BRAF, HSP90AA1, IGF1R, KRAS, MAPK3, PGR, PIK3CA, PIK3CD, PIK3R1, PIK3R2
Phospholipase D signaling pathway	7	149	AKT1, AKT3, FYN, GNAS, GRM3, INSR, KRAS, MAP2K2, MAPK3, MTOR, PDGFRA, PDGFRB, PIK3CA, PIK3CD, PIK3R1, PIK3R2, PLCG2, SYK, TSC1, TSC2
JAK-STAT signaling pathway	7	168	AKT1, AKT3, CCND2, CREBBP, CSF3R, EP300, IL10, IL7R, JAK1, JAK3, MCL1, MPL, MTOR, PDGFRA, PDGFRB, PIK3CA, PIK3CD, PIK3R1, PIK3R2, SOCS1, STAT4
Viral carcinogenesis	7	205	JUN, CASP8, CCND2, CDK4, CDKN2A, CHD4, CREBBP, EP300, JAK1, JAK3, KRAS, LYN, MAPK3, PIK3CA, PIK3CD, PIK3R1, PIK3R2, RB1, REL, SYK, TP53, TRAF2
Bacterial invasion of epithelial cells	7	78	CBL, CDH1, CTNNA1, CTNNB1, MET, PIK3CA, PIK3CD, PIK3R1, PIK3R2
Aldosterone-regulated sodium reabsorption	7	38	INSR, IRS1, KRAS, MAPK3, PIK3CA, PIK3CD, PIK3R1, PIK3R2
Prolactin signaling pathway	7	71	AKT1, AKT3, CCND2, ESR1, KRAS, MAP2K2, MAPK3, PIK3CA, PIK3CD, PIK3R1, PIK3R2, SOCS1

Estrogen signaling pathway	6	139	JUN, AKT1, AKT3, ESR1, GNAS, HSP90AA1, KRAS, MAP2K2, MAPK3, NCOA3, PGR, PIK3CA, PIK3CD, PIK3R1, PIK3R2, RARA
Basal cell carcinoma	6	63	APC, AXIN1, AXIN2, CTNNB1, GLI1, PTCH1, SMO, SUFU, TCF7L2, TP53
Cell cycle	6	158	ABL1, ATM, ATR, ATRX, CCND2, CDK4, CDKN2A, CHEK2, CREBBP, E2F3, EP300, PRKDC, RAD21, RB1, SMAD2, SMAD3, SMC3, STAG1, STAG2, TP53
AGE-RAGE signaling pathway in diabetic complications	6	101	JUN, AKT1, AKT3, CDK4, KRAS, MAPK3, PIK3CA, PIK3CD, PIK3R1, PIK3R2, PLCG2, SMAD2, SMAD3, TGFBR1, TGFBR2, VEGFA
C-type lectin receptor signaling pathway	6	105	JUN, AKT1, AKT3, CASP8, IKBKE, IL10, KRAS, MALT1, MAP3K14, MAPK3, PAK1, PIK3CA, PIK3CD, PIK3R1, PIK3R2, PLCG2, SYK
Chagas disease	6	103	JUN, AKT1, AKT3, CALR, CASP8, FAS, GNAS, IL10, MAP2K4, MAPK3, PIK3CA, PIK3CD, PIK3R1, PIK3R2, SMAD2, TGFBR1, TGFBR2
Calcium signaling pathway	6	254	ERBB2, ERBB3, ERBB4, FGFR1, FGFR2, FGFR3, FGFR4, FLT1, FLT4, GNAS, HGF, MET, MST1, MST1R, NTRK1, NTRK3, PDGFRA, PDGFRB, PLCG2, RET, VEGFA
T cell receptor signaling pathway	6	122	JUN, AKT1, AKT3, CARD11, CDK4, CTLA4, FYN, IL10, KRAS, MALT1, MAP2K2, MAP3K14, MAPK3, PAK1, PAK3, PIK3CA, PIK3CD, PIK3R1, PIK3R2
Epstein-Barr virus infection	6	205	JUN, AKT1, AKT3, B2M, BTK, CALR, CASP8, CCND2, CDK4, E2F3, FAS, IKBKE, JAK1, JAK3, LYN, MAP2K4, MAP3K14, PIK3CA, PIK3CD, PIK3R1, PIK3R2, PLCG2, RB1, SYK, TP53, TRAF2
Regulation of actin cytoskeleton	6	232	AKT1, AKT3, APC, ARAF, BRAF, FGFR1, FGFR2, FGFR3, FGFR4, KRAS, MAP2K2, MAPK3, PAK1, PAK3, PDGFRA, PDGFRB, PIK3CA, PIK3CD, PIK3R1, PIK3R2
GnRH secretion	6	65	AKT1, AKT3, KRAS, MAP2K2, MAPK3, PIK3CA, PIK3CD, PIK3R1, PIK3R2
Shigellosis	5	253	JUN, AKT1, AKT3, ATM, MALT1, MAPK3, MTOR, PIK3CA, PIK3CD, PIK3R1, PIK3R2, PLCG2, RPS6KB1, RPS6KB2, RPTOR, TP53, TRAF2, U2AF1

Chemical carcinogenesis - reactive oxygen species	5	227	JUN, ABL1, ABL2, AKT1, AKT3, ARAF, BRAF, HGF, KEAP1, KRAS, MAP2K2, MAP2K4, MAP3K14, MAPK3, MET, NFE2L2, PIK3CA, PIK3CD, PIK3R1, PIK3R2, PTEN, SDHA, SDHC, SDHD, VEGFA
Lipid and atherosclerosis	5	216	JUN, AKT1, AKT3, CASP8, FAS, HSP90AA1, IKBKE, KRAS, LYN, MAP2K4, MAPK3, NFE2L2, PIK3CA, PIK3CD, PIK3R1, PIK3R2, TP53, TRAF2
Autophagy - animal	5	169	AKT1, AKT3, IGF1R, IRS1, IRS2, KRAS, MAP2K2, MAPK3, MTOR, PIK3CA, PIK3CD, PIK3R1, PIK3R2, PTEN, RPS6KB1, RPS6KB2, RPTOR, STK11, TSC1, TSC2
mTOR signaling pathway	5	158	AKT1, AKT3, BRAF, FLCN, IGF1R, INSR, IRS1, KRAS, MAP2K2, MAPK3, MTOR, PIK3CA, PIK3CD, PIK3R1, PIK3R2, PTEN, RPS6KB1, RPS6KB2, RPTOR, STK11, TSC1, TSC2
AMPK signaling pathway	5	122	AKT1, AKT3, IGF1R, INSR, IRS1, IRS2, MTOR, PIK3CA, PIK3CD, PIK3R1, PIK3R2, RPS6KB1, RPS6KB2, RPTOR, STK11, TSC1, TSC2
Adherens junction	5	93	CDH1, CREBBP, CTNNA1, CTNNB1, EP300, ERBB2, FGFR1, FYN, IGF1R, INSR, MAPK3, MET, SMAD3, TCF7L2, TGFBR1, TGFBR2, YES1
Relaxin signaling pathway	5	130	JUN, AKT1, AKT3, GNAS, KRAS, MAP2K2, MAP2K4, MAPK3, PIK3CA, PIK3CD, PIK3R1, PIK3R2, SMAD2, TGFBR1, TGFBR2, VEGFA
Insulin resistance	5	109	AKT1, AKT3, INSR, IRS1, IRS2, MTOR, PIK3CA, PIK3CD, PIK3R1, PIK3R2, PTEN, RPS6KB1, RPS6KB2
Fc gamma R-mediated phagocytosis	5	99	AKT1, AKT3, LYN, MAPK3, PAK1, PIK3CA, PIK3CD, PIK3R1, PIK3R2, PLCG2, RPS6KB1, RPS6KB2, SYK
Chemical carcinogenesis - receptor activation	5	217	JUN, AKT1, AKT3, AR, BCL6, ESR1, GNAS, HSP90AA1, IKBKE, KLF4, KRAS, MAP2K2, MAPK3, MTOR, NOTCH2, PGR, PIK3CA, PIK3CD, PIK3R1, PIK3R2, RB1, RPS6KB1, RPS6KB2, VEGFA, XIAP
Polycomb repressive complex	4	83	ASXL1, ASXL2, BCOR, BCORL1, CREBBP, EP300, MGA
Diabetic cardiomyopathy	4	205	AKT1, AKT3, INSR, IRS1, MTOR, PARP1, PIK3CA, PIK3CD, PIK3R1, PIK3R2, PTEN, SDHA, SDHC, SDHD, SMAD2, SMAD3, TGFBR1, TGFBR2

Human immunodeficiency virus 1 infection	4	214	JUN, AKT1, AKT3, ATM, ATR, B2M, CALR, CASP8, FAS, KRAS, MAP2K2, MAPK3, MTOR, PAK1, PAK3, PIK3CA, PIK3CD, PIK3R1, PIK3R2, PLCG2, RPS6KB1, RPS6KB2, TRAF2
Insulin signaling pathway	4	138	AKT1, AKT3, ARAF, BRAF, CBL, INSR, IRS1, IRS2, KRAS, MAP2K2, MAPK3, MTOR, PIK3CA, PIK3CD, PIK3R1, PIK3R2, RPS6KB1, RPS6KB2, RPTOR, SOCS1, TSC1, TSC2
Axon guidance	4	184	ABL1, EPHA3, EPHA5, EPHA7, EPHB1, FYN, KRAS, MAPK3, MET, PAK1, PAK3, PIK3CA, PIK3CD, PIK3R1, PIK3R2, PLCG2, PTCH1, RASA1, SLIT2, SMO
Th17 cell differentiation	4	109	JUN, GATA3, HSP90AA1, IRF4, JAK1, JAK3, MAPK3, MTOR, RARA, RUNX1, SMAD2, SMAD3, TGFBR1, TGFBR2
Carbohydrate digestion and absorption	4	52	AKT1, AKT3, PIK3CA, PIK3CD, PIK3R1, PIK3R2
Toll-like receptor signaling pathway	4	109	JUN, AKT1, AKT3, CASP8, IKBKE, JAK1, MAP2K2, MAP2K4, MAPK3, PIK3CA, PIK3CD, PIK3R1, PIK3R2
TNF signaling pathway	4	119	JUN, AKT1, AKT3, BIRC3, CASP8, FAS, MAP2K4, MAP3K14, MAPK3, PIK3CA, PIK3CD, PIK3R1, PIK3R2, RPS6KA4, TRAF2, XIAP
Inositol phosphate metabolism	3	78	PIK3C2B, PIK3C2G, PIK3CA, PIK3CD, PLCG2, PTEN
Phosphatidylinositol signaling system	3	98	PIK3C2B, PIK3C2G, PIK3CA, PIK3CD, PIK3R1, PIK3R2, PLCG2, PTEN
Non-alcoholic fatty liver disease	3	157	JUN, AKT1, AKT3, CASP8, FAS, INSR, IRS1, IRS2, PIK3CA, PIK3CD, PIK3R1, PIK3R2, SDHA, SDHC, SDHD, TRAF2
Amoebiasis	3	103	GNAS, IL10, PIK3CA, PIK3CD, PIK3R1, PIK3R2
Platelet activation	3	126	AKT1, AKT3, BTK, FYN, GNAS, LYN, MAPK3, PIK3CA, PIK3CD, PIK3R1, PIK3R2, PLCG2, SYK
Apelin signaling pathway	3	140	AKT1, AKT3, CDH1, KRAS, MAP2K2, MAPK3, MTOR, NOTCH3, RPS6KB1, RPS6KB2, SMAD2, SMAD3, TGFBR1
Inflammatory mediator regulation of TRP channels	3	99	GNAS, NTRK1, PIK3CA, PIK3CD, PIK3R1, PIK3R2, PLCG2
Cushing syndrome	3	155	APC, AXIN1, AXIN2, BRAF, CDK4, CDKN2A, CTNNB1, E2F3, FH, GNAS, HSD3B1, KMT2A, MAP2K2, MAPK3, RB1, TCF7L2
Notch signaling pathway	3	62	CREBBP, EP300, NOTCH1, NOTCH2, NOTCH3, NOTCH4, SPEN

Cholinergic synapse	3	116	AKT1, AKT3, FYN, KRAS, MAPK3, PIK3CA, PIK3CD, PIK3R1, PIK3R2
Yersinia infection	3	138	JUN, AKT1, AKT3, IL10, MAP2K2, MAP2K4, MAPK3, PIK3CA, PIK3CD, PIK3R1, PIK3R2, TRAF2
Osteoclast differentiation	3	143	JUN, AKT1, AKT3, BTK, CSF1R, FYN, JAK1, MAP3K14, MAPK3, PIK3CA, PIK3CD, PIK3R1, PIK3R2, PLCG2, SOCS1, SYK, TGFBR1, TGFBR2, TRAF2
Salmonella infection	2	251	JUN, AKT1, AKT3, BIRC3, CASP8, CTNNB1, HSP90AA1, KIF5B, MAP2K2, MAP2K4, MAPK3, PAK1, PAK3, PIK3C2B, PIK3C2G, PIK3CA, PIK3CD, TCF7L2, TRAF2
Spinocerebellar ataxia	2	144	AKT1, AKT3, CIC, MTOR, MYOD1, PIK3CA, PIK3CD, PIK3R1, PIK3R2, TRAF2
Th1 and Th2 cell differentiation	2	93	JUN, GATA3, JAK1, JAK3, MAPK3, NOTCH1, NOTCH2, NOTCH3, STAT4
Influenza A	2	173	AKT1, AKT3, CASP8, CDK4, CREBBP, EP300, FAS, IKBKE, JAK1, MAP2K2, MAPK3, PIK3CA, PIK3CD, PIK3R1, PIK3R2, XPO1
Natural killer cell mediated cytotoxicity	2	134	ARAF, BRAF, FAS, FYN, KRAS, MAP2K2, MAPK3, PAK1, PIK3CA, PIK3CD, PIK3R1, PIK3R2, PLCG2, SYK
cAMP signaling pathway	2	226	JUN, AKT1, AKT3, BRAF, CREBBP, EP300, GLI1, GNAS, MAP2K2, MAPK3, PAK1, PIK3CA, PIK3CD, PIK3R1, PIK3R2, PTCH1, SOX9, TSHR
Mitophagy - animal	2	105	JUN, KRAS, PRKN, TFE3, TP53, TRAF2
Gap junction	2	92	GNAS, KRAS, MAP2K2, MAPK3, PDGFRA, PDGFRB
Leukocyte transendothelial migration	2	116	CTNNA1, CTNNB1, PIK3CA, PIK3CD, PIK3R1, PIK3R2, PLCG2
Hippo signaling pathway	2	157	APC, AXIN1, AXIN2, BIRC3, CCND2, CDH1, CTNNA1, CTNNB1, LATS2, SMAD2, SMAD3, TCF7L2, TGFBR1, TGFBR2
Ovarian steroidogenesis	1	52	GNAS, HSD3B1, IGF1R, INSR
Prion disease	1	278	FYN, KIF5B, MAPK3, NOTCH1, PIK3CA, PIK3CD, PIK3R1, PIK3R2, SDHA, SDHC, SDHD
Malaria	1	50	HGF, IL10, MET

Neutrophil extracellular trap formation	1	196	AKT1, AKT3, MAP2K2, MAPK3, MTOR, PIK3CA, PIK3CD, PIK3R1, PIK3R2, PLCG2, SYK
Pathogenic Escherichia coli infection	1	203	ABL1, CASP8, FAS, FYN, JUN, MAPK3, PAK1, PAK3, TRAF2
Viral myocarditis	1	70	ABL1, ABL2, CASP8, FYN
Melanogenesis	1	101	CREBBP, CTNNB1, EP300, GNAS, KRAS, MAP2K2, MAPK3, TCF7L2
RIG-I-like receptor signaling pathway	1	72	CASP8, IKBKE, MAP3K1, TRAF2
Cell adhesion molecules	1	160	CD276, CDH1, CTLA4, ICOSLG, NEGR1, PTPRD, PTPRS, VTCN1
Parathyroid hormone synthesis, secretion and action	1	11	ARAF, BRAF, FGFR1, GATA3, GNAS, MAPK3
Parkinson disease	1	271	DAXX, GNAS, KEAP1, KIF5B, NFE2L2, PRKN, SDHA, SDHC, SDHD, SNCAIP, TP53
Inflammatory bowel disease	1	66	JUN, GATA3, IL10, SMAD2, SMAD3, STAT4
Autoimmune thyroid disease	1	54	CTLA4, FAS, IL10, TSHR
Ferroptosis	1	42	TFRC, TP53
Nucleotide excision repair	1	63	ERCC1, ERCC2, ERCC3, ERCC5, POLD1, POLE
Hedgehog signaling pathway	1	56	CCND2, CSNK1A1, GLI1, PTCH1, SMO, SUFU
Thermogenesis	1	235	ARID1A, ARID1B, FGFR1, GNAS, KRAS, MTOR, RPS6KB1, RPS6KB2, RPTOR, SDHA, SDHC, SDHD, TSC1, TSC2
Lysine degradation	1	63	DOT1L, KMT2A, NSD1, SETD2
Intestinal immune network for IgA production	1	50	ICOSLG, IL10, MAP3K14
Long-term potentiation	1	67	ARAF, BRAF, CREBBP, EP300, KRAS, MAP2K2, MAPK3
Long-term depression	1	60	ARAF, BRAF, GNAS, IGF1R, KRAS, LYN, MAP2K2, MAPK3
Alzheimer disease	1	391	AKT1, AKT3, APC, ARAF, AXIN1, AXIN2, BRAF, CASP8, CSNK1A1, CTNNB1, FAS, INSR, IRS1, IRS2, KIF5B, KRAS, MAP2K2, MAPK3, MTOR, PIK3CA, PIK3CD, PIK3R1, PIK3R2, SDHA, SDHC, SDHD, TRAF2

Supplementary Table S3. Univariate Cox regression analysis of local progression-free survival according to pathway mutations

Variable	Univariate		
	HR	95% Cl	p-value
Central carbon metabolism in cancer	0.46	0.19 - 1.11	0.085
PI3K-Akt signaling pathway	0.23	0.10 - 0.56	0.001
Prostate cancer	0.50	0.21 - 1.16	0.106
Non-small cell lung cancer	0.38	0.16 - 0.90	0.028
Breast cancer	0.83	0.37 - 1.86	0.646
EGFR tyrosine kinase inhibitor resistance	0.35	0.15 - 0.84	0.019
Endocrine resistance	0.73	0.32 - 1.66	0.460
Glioma	0.38	0.15 - 0.93	0.035
Pancreatic cancer	0.61	0.26 - 1.43	0.257
Melanoma	0.90	0.40 - 2.03	0.794
Platinum drug resistance	0.70	0.30 - 1.64	0.413
Proteoglycans in cancer	0.42	0.17 - 1.03	0.057
MicroRNAs in cancer	0.51	0.21 - 1.21	0.126
Colorectal cancer	0.66	0.28 - 1.56	0.347
Ras signaling pathway	0.38	0.15 - 0.97	0.043
Thyroid hormone signaling pathway	0.26	0.10 - 0.73	0.010
Hepatocellular carcinoma	0.97	0.43 - 2.19	0.943
Gastric cancer	0.80	0.35 - 1.84	0.599
MAPK signaling pathway	0.93	0.40 - 2.17	0.871
Endometrial cancer	0.72	0.31 - 1.70	0.456
Chronic myeloid leukemia	0.32	0.11 - 0.87	0.026
Signaling pathways regulating pluripotency of stem cells	0.14	0.04 - 0.49	0.002
Renal cell carcinoma	0.42	0.16 - 1.07	0.070
Human papillomavirus infection	0.70	0.29 - 1.70	0.434
Small cell lung cancer	0.56	0.22 - 1.42	0.221
Longevity regulating pathway	0.48	0.18 - 1.30	0.148
Cellular senescence	0.55	0.20 - 1.49	0.241
Neurotrophin signaling pathway	0.24	0.08 - 0.73	0.012
ErbB signaling pathway	0.19	0.06 - 0.68	0.010
FoxO signaling pathway	0.36	0.12 - 1.06	0.064
Human T-cell leukemia virus 1 infection	0.86	0.34 - 2.18	0.754
Longevity regulating pathway - multiple species	0.05	0.01 - 0.41	0.005
Acute myeloid leukemia	0.11	0.03 - 0.50	0.004
Sphingolipid signaling pathway	0.47	0.17 - 1.27	0.136
Apoptosis	0.70	0.28 - 1.78	0.456
Kaposi sarcoma-associated herpesvirus infection	0.20	0.06 - 0.71	0.012
HIF-1 signaling pathway	0.13	0.03 - 0.58	0.007

Hepatitis B	0.54	0.18 - 1.59	0.264
Rap1 signaling pathway	0.72	0.27 - 1.94	0.514
Fanconi anemia pathway	0.66	0.24 - 1.80	0.413
VEGF signaling pathway	0.15	0.03 - 0.66	0.012
B cell receptor signaling pathway	0.06	0.01 - 0.46	0.007
Fc epsilon RI signaling pathway	0.06	0.01 - 0.46	0.007
Homologous recombination	0.67	0.22 – 2.00	0.469
p53 signaling pathway	1.55	0.59 - 4.04	0.374
Measles	0.41	0.12 - 1.40	0.157
PD-L1 expression and PD-1 checkpoint pathway in cancer	0.33	0.10 - 1.11	0.073
Focal adhesion	0.67	0.23 - 1.97	0.466
Human cytomegalovirus infection	0.54	0.18 - 1.60	0.267
Transcriptional misregulation in cancer	1.04	0.38 - 2.85	0.933
Regulation of lipolysis in adipocytes	0.17	0.04 - 0.74	0.018
Hepatitis C	0.56	0.19 - 1.67	0.299
Thyroid cancer	0.92	0.34 - 2.46	0.864
Choline metabolism in cancer	0.20	0.05 - 0.88	0.033
Type II diabetes mellitus	0.08	0.01 - 0.65	0.017
Prolactin signaling pathway	0.08	0.01 - 0.58	0.013
Bladder cancer	1.15	0.38 - 3.45	0.803
Calcium signaling pathway	1.18	0.46 - 2.99	0.730
Aldosterone-regulated sodium reabsorption	0.13	0.02 - 0.99	0.049
Progesterone-mediated oocyte maturation	0.62	0.18 - 2.07	0.435
Estrogen signaling pathway	0.12	0.02 - 0.88	0.037
Fluid shear stress and atherosclerosis	0.75	0.26 - 2.20	0.599
Bacterial invasion of epithelial cells	0.39	0.09 - 1.65	0.199
Chemical carcinogenesis - receptor activation	0.09	0.01 - 0.71	0.023
Phospholipase D signaling pathway	0.27	0.06 - 1.16	0.079
JAK-STAT signaling pathway	0.31	0.07 - 1.33	0.115
Cell cycle	1.84	0.61 - 5.54	0.276
AGE-RAGE signaling pathway in diabetic complications	0.46	0.11 - 1.95	0.291
Epstein-Barr virus infection	0.50	0.12 - 2.14	0.351
Chemical carcinogenesis - reactive oxygen species	1.19	0.40 - 3.50	0.753
Viral carcinogenesis	1.19	0.35 - 4.08	0.779
Inositol phosphate metabolism	0.18	0.02 - 1.34	0.094
AMPK signaling pathway	0.34	0.08 - 1.48	0.151
Insulin resistance	0.35	0.08 - 1.51	0.159
C-type lectin receptor signaling pathway	0.21	0.03 - 1.57	0.129
Chagas disease	0.23	0.03 - 1.69	0.148
T cell receptor signaling pathway	0.26	0.04 - 1.95	0.191
Axon guidance	0.17	0.02 - 1.29	0.087

Regulation of actin cytoskeleton	0.44	0.10 - 1.87	0.265
GnRH secretion	0.22	0.03 - 1.61	0.135
Notch signaling pathway	0.10	0.01 - 0.81	0.031
Basal cell carcinoma	1.67	0.56 - 5.01	0.361
Lipid and atherosclerosis	0.46	0.11 - 1.98	0.298
Autophagy - animal	0.48	0.11 - 2.03	0.316
mTOR signaling pathway	0.49	0.11 - 2.08	0.332
Adherens junction	1.00	0.23 - 4.33	0.998
Relaxin signaling pathway	0.32	0.04 - 2.42	0.272
Fc gamma R-mediated phagocytosis	0.25	0.03 - 1.90	0.182
